# Impaired Exercise Capacity and Mortality Risk in Adults With Congenital Heart Disease

**DOI:** 10.1016/j.jacadv.2023.100422

**Published:** 2023-07-28

**Authors:** Anna Wikner, Anette Sandström, Daniel Rinnström, Urban Wiklund, Christina Christersson, Mikael Dellborg, Niels Erik Nielsen, Peder Sörensson, Ulf Thilén, Bengt Johansson, Camilla Sandberg

**Affiliations:** a Department of Public Health and Clinical Medicine, Umeå University, Umeå, Sweden; b Department of Surgery and Perioperative Sciences, Umeå University, Umeå, Sweden; c Department of Radiation Sciences, Radiation Physics, Biomedical Engineering, Umeå University, Umeå, Sweden; d Department of Medical Sciences, Cardiology Uppsala University, Uppsala, Sweden; e Department of Molecular and Clinical Medicine, University of Gothenburg, Gothenburg, Sweden; f Department of Medical and Health Sciences, Linkoping University, Linköping, Sweden; g Department of Medicine, Karolinska Institutet, Stockholm, Sweden; h Department of Cardiology, Clinical Sciences, Skane University Hospital, Lund, Sweden; i Department of Community Health and Rehabilitation, Umeå University, Umeå, Sweden

**Keywords:** aerobic exercise capacity, congenital heart disease, exercise test, mortality, outcome

## Abstract

**Background:**

An association between impaired exercise capacity and risk of mortality has been reported among adults with congenital heart disease (CHD). Over the years, treatment methods have improved and may influence outcome. Hence, we report data from a national cohort reflecting a contemporary population.

**Objectives:**

The purpose of this study was to investigate the association between exercise capacity (workload) and mortality in a large registry-based cohort.

**Methods:**

Data on exercise capacity using cycle ergometer were retrieved from the national registry of CHD. The association between predicted exercise capacity (%EC_pred_) and mortality was analyzed using Cox regression.

**Results:**

In total, 3,721 adults (>18 years, 44.6% women) with CHD were included. The median age was 27.0 years (IQR: 20.8-41.0 years) and mean %EC_pred_ was 77% ± 20%. Over a mean follow-up of 9.4 ± 6.0 years, there were 214 (5.8%) deaths. The Multivariable Cox regression model showed that moderately and severely impaired exercise capacity (50-<70 %EC_pred_: HR: 2.1, 95% CI: 1.4-3.2, *P* < 0.001, and <50 %EC_pred_: HR: 3.5, 95% CI: 2.1-6.0, *P* < 0.001) and CHD complexity were associated with higher mortality (moderate complexity: HR: 1.9 95% CI: 1.2-3.0, *P* = 0.003, great complexity: HR: 2.3 95% CI: 1.3-4.2, *P* = 0.008) when adjusted for New York Heart Association class, physical activity, cardiovascular medication, sex, impaired systemic ventricular function, and age.

**Conclusions:**

Impaired exercise capacity and CHD complexity are independently associated with all-cause mortality in patients with CHD. Exercise capacity is an easily accessible variable that may be a useful tool for risk assessment in adult patients with CHD, but this needs confirmation in prospective studies.

Adults with congenital heart disease (CHD) are a constantly growing population thanks to considerable progress in medical and surgical treatments. Nowadays, 90% to 97% of infants born with CHD survive into adulthood, and the adult population exceeds the number of children.^1^, ^2^, ^3^ Nevertheless, patients with CHD still have a reduced lifespan compared with the general population.^1^^,^^4^^,^^5^ Therefore, it is important to find tools to identify patients at risk of worse outcome, and if possible, to enable early intervention.

Impaired aerobic exercise capacity is a common finding in persons with CHD. Patients with complex lesions^6^ generally have a more pronounced impairment, but there is a wide range in exercise capacity within each diagnostic group.^7^, ^8^, ^9^ Furthermore, reduced exercise capacity is known to be associated with a worse prognosis in the general population,^10^^,^^11^ acquired heart failure,^12^ and CHD.^8^ As early as 2006, Diller et al^8^ showed an association between impaired peak oxygen uptake and hospitalization/mortality, as a composite endpoint, where hospitalizations were the dominating outcome. Since then, treatment methods have improved further resulting in increased survival.^1^ Furthermore, previous reports regarding the association between different measures of exercise capacity and outcome in CHD are mainly derived from highly specialized and single centers.^8^ This may present a risk of a patient selection favoring more severe cases. A majority of these reports are focusing on subgroups of lesions, eg, tetralogy of Fallot.^8^^,^^13^, ^14^, ^15^, ^16^, ^17^ Also, studies regarding the association between peak exercise capacity in terms of workload and mortality, as primary outcome, are scarce.

Therefore, the primary aim was to investigate the association between percent of predicted peak exercise capacity and mortality in adult patients with CHD in a nationwide population. The secondary aim was to identify predictors of mortality.

## Methods

### The SWEDCON Registry

The SWEDCON (Swedish Registry of Congenital Heart Disease)^2^ covers all 7 health regions in Sweden. The register contains data on demographics, and medical and surgical data such as diagnosis, interventions, and cardiovascular medication. Data encompass cardiovascular symptoms, exercise test results, NYHA functional class, electrocardiogram, self-reported physical activity level, and echocardiography including biventricular function. In this study data on ventricular function was grouped binary—normal and impaired (slightly, moderately, or severely impaired). Clinical data used in analysis were retrieved from the clinical visit closest in time to the exercise test, no longer than 2 years apart.

Data on all-cause mortality are received from the national population register at monthly reconciliations.^2^ The validity of data is reported as high.^18^

The study was approved by the Regional Ethics Review Board in Umeå, Sweden (Dnr 08-218 M, Dnr 2012-445-32 M, Dnr 2019-04745).

### Patient selection

**Figure 1:**
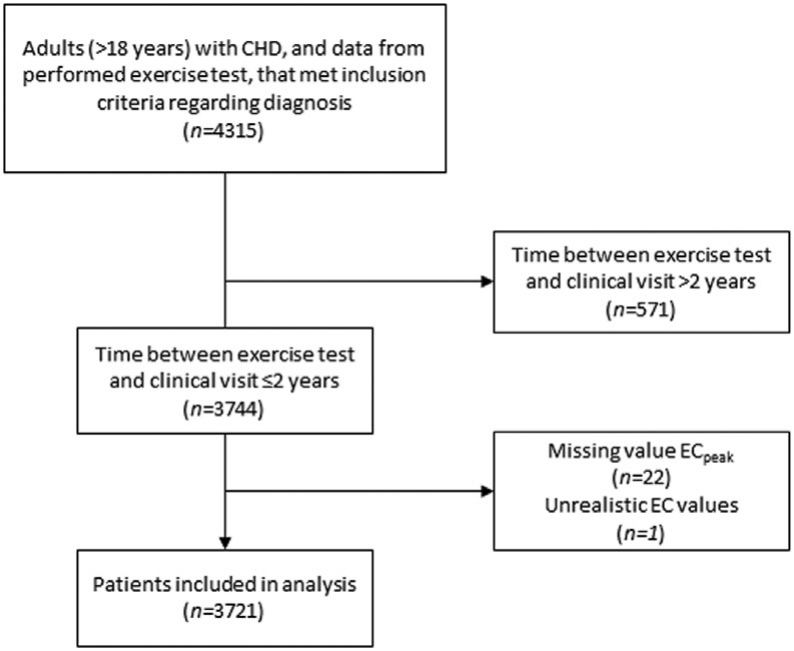
**Patient Selection** Flow chart over inclusion process. CHD = congenital heart disease; EC = exercise capacity; EC_peak_ = peak exercise capacity.

Data were extracted from SWEDCON on October 6, 2017. There were 4,315 adult patients (>18 years of age) with data on exercise capacity that met inclusion criteria regarding the following CHD diagnoses: congenital aortic valve disease, coarctation of the aorta (CoA), atrial septal defect, ventricular septal defect, tetralogy of Fallot, pulmonary atresia with intact ventricular septum or with ventricular septal defect, double outlet right ventricle, congenital corrected transposition of the great arteries, dextro-transposition of the great arteries corrected with arterial switch or a Rastelli procedure, dextro-transposition of the great arteries corrected with a Senning/Mustard procedure, atrioventricular septal defect, patent ductus arteriosus, Ebsteins anomaly, pulmonary stenosis, truncus arteriosus, and Fontan circulation. In addition, a group was included with various congenital heart defects (miscellaneous) not possible to classify into one of the aforementioned groups. Data on exercise capacity were retrieved from exercise tests performed on cycle ergometers. In the case of multiple exercise tests, only data from the first test were included in the analysis. Furthermore, patients without a clinical visit within 2 years from the exercise test were excluded from analysis, thus a total of 3,721 were included in analysis (Figure 1).

### Peak exercise test

Evaluation of aerobic exercise capacity was performed on a cycle ergometer using standardized protocols. The incremental increase of workload is usually 10 to 20 W/min and conventional criteria of test termination were applied.^19^ The exercise tests were performed between June 1, 1990, and September 19, 2017.

### Predicted peak exercise capacity

Predicted peak workload was calculated according to sex, age, and height by using the equation proposed by Brudin et al.^20^ The percentage of predicted peak workload was calculated according to the achieved peak workload (W_peak_/W_pred_). The percentage of predicted peak workload will henceforth be referred to as the percent of predicted exercise capacity (%EC_pred_). When data on height were missing, imputation of height was performed using the means for sex in 5-year age groups. After imputation of height (n = 139), it was possible to calculate the percent of predicted workload for all 3,721 patients included in the study (Figure 1).

The %EC_pred_ was first categorized according to national standards, ie, good (≥120 %EC_pred_), normal (75-120 %EC_pred_), borderline (70-75 %EC_pred_), moderately impaired (50-70 %EC_pred_), and severely impaired (<50 %EC_pred_).^21^ Thereafter, the categories good, normal, and borderline exercise capacity were merged into 1 category, herein referred to as normal exercise capacity (≥70 %EC_pred_).

### Statistical analysis

Statistical analyses were performed using IBM SPSS statistics 26.0-27.0 (IBM).

Data were assessed for normality and presented as mean ± SD or median (IQR). Frequencies were presented as numbers with percentages. For comparison, the chi-square test was used for categorical variables, and the Student’s *t*-test or Mann-Whitney *U* test was used for analysis of continuous variables. The probability of survival was assessed using Kaplan-Meier curves and tested using log-rank statistics. As the conditions of proportional hazards were fulfilled, the association between exercise capacity and mortality was assessed using univariable and multivariable Cox regression analyses. Covariates introduced in the Cox regression model, besides the 3 categories of %EC_pred_, were complexity of CHD (simple, moderate, and great complexity according to main diagnosis),^6^ NYHA functional class, cardiovascular medication, level of physical activity, left ventricular function, age at exercise test, and sex. When classifying the groups of diagnoses according to complexity, the group of patients with miscellaneous diagnoses was excluded since they were not possible to classify. Thus, the patients with miscellaneous diagnoses (n = 376) were not included in the multivariable Cox regression analysis. Covariates were chosen based on theoretical estimations along with results from univariable analyses. The model was evaluated in both manual (enter) forward and backward manner and presented as an initial and a final reduced model. The numbers included in the model, as well as multicollinearity, were monitored during each step. The presence of cardiovascular symptoms was statistically important in univariable analysis but was not included in the multivariable model due to its strong correlation with NYHA functional class (r = 0.54, *P* < 0.001). Time to event or censoring was set between exercise test and date of death or date of data extraction (October 6, 2017). In order to evaluate the additive value of including %ECpred in the regression models, logistic regression analyses were performed using the same independent variables as described above and with outcome as death at 5 and 10 years after performed exercise test. The area under the curve (AUC) was calculated for both models with and without %ECpred as an independent variable. To compare patients included in the present report, that is, patients with data on exercise capacity, with patients without exercise test, a dropout analysis regarding mortality rate, diagnosis group, and sex was performed. In all analyses, the null hypothesis was rejected on *P* values <0.05.

## Results

### Study population

In total, 3,721 patients were included in the analysis. The median age at the performed exercise test was 27.0 years (IQR: 20.8-41.0) and 44.6% were women (Table 1). The most common diagnoses were congenital aortic valve disease (17.8%) and CoA (15.1%) (Supplemental Table 1). Additionally, when categorized according to complexity, diagnosis of moderate complexity was most common (52%). The majority of patients were in NYHA functional class I (Table 1).

**Table 1:** Patient Characteristics

	All patients (n = 3,721)	Deceased (n = 214)	Alive (n = 3,507)	*P* Value
Female	1,658 (44.6)	93 (43.5)	1,565 (44.6)	0.7
Age y	27.0 (20.8, 41.0)	44.8 (28.6, 57.3)	26.4 (20.7, 40.0)	**<0.001**
Smoking (n = 3,458)				0.2
Yes	363 (10.5)	27 (14.4)	336 (10.3)	
Previous	180 (5.2)	9 (4.8)	171 (5.2)	
Physical exercise (n = 3,372)				**<0.001**
None	1,461 (43.3)	113 (61.7)	1,348 (42.3)	
<3 h/wk	1,136 (33.7)	55 (30.1)	1,081 (33.9)	
>3 h/wk	775 (23.0)	15 (8.2)	760 (23.8)	
Cardiovascular medication^a^ (n = 3,664)				
Yes	1,052 (28.7)	97 (45.5)	955 (27.7)	**<0.001**
Pacemaker/ICD (n = 3,652)				
Yes	152 (4.2)	12 (5.7)	140 (4.1)	0.3
NYHA functional class (n = 3,293)				**<0.001**
I	2,635 (80.0)	106 (57.3)	2,529 (81.4)	
II	549 (16.7)	55 (29.7)	494 (15.9)	
III	106 (3.2)	23 (12.4)	83 (2.7)	
IV	3 (0.1)	1 (0.5)	2 (0.1)	
Symptoms^b^ (n = 3,681)				
Yes	1,096 (29.8)	95 (45.5)	1,001 (28.8)	**<0.001**
Systemic ventricular function (n = 3,442)				
Impaired	388 (11.3)	42 (21.5)	346 (10.7)	**<0.001**
Subpulmonary ventricular function (n = 3,226)				
Impaired	325 (10.1)	25 (14.3%)	300 (9.8)	**0.06**
CHD complexity (n = 3,405)				**0.02**
Simple	950 (27.9)	69 (34.7)	881 (27.5)	
Moderate complexity	1,756 (51.6)	83 (41.7)	1,673 (52.2)	
Great complexity	699 (20.5)	47 (23.6)	652 (20.3)	
%EC_pred_	77 ± 20	64 ± 21	78 ± 20	**<0.001**

Values are n (%), median (25th, 75th quartile), and mean ± SD. **Bold** values denote *P* < 0.05 between deceased and alive.

CHD = congenital heart disease; %EC_pred_ = percent of predicted peak exercise capacity; ICD = implantable cardioverter-defibrillator.

a Cardiovascular medication, eg, digoxin, anticoagulant and antiplatelet therapies, anti-arrhythmia class I-IV and diuretics.

b Symptoms, ie, syncope, fatigue, palpitations, dyspnea, chest pain, and edema.

### Aerobic exercise capacity

**Figure 2:**
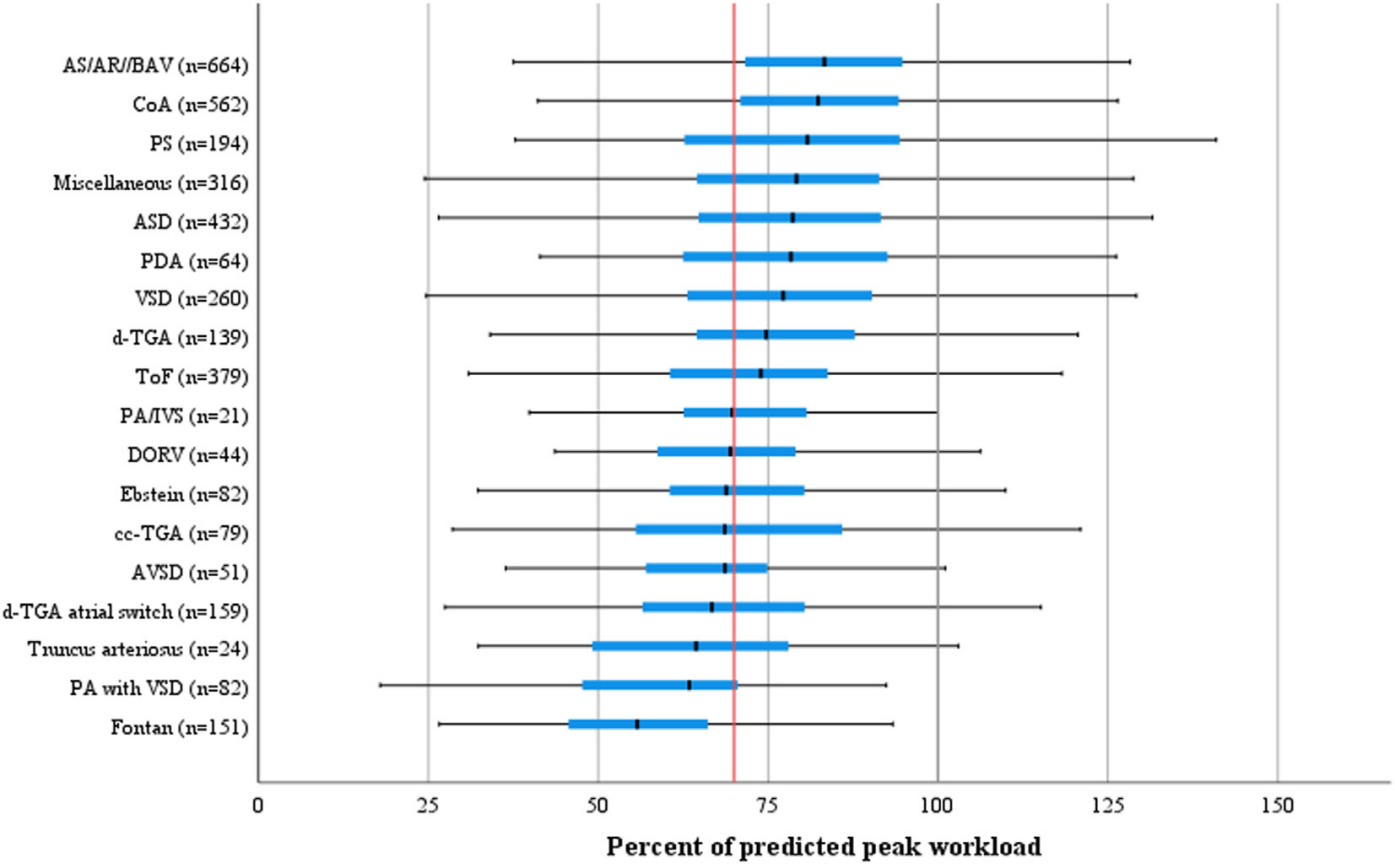
**Percent of Predicted Peak Exercise Capacity According to Diagnosis** Boxplot showing percent of predicted peak exercise capacity according to congenital heart lesion. The **black and red vertical lines** indicate 100% and 70% percent of predicted exercise capacity (workload), respectively. AR = aortic regurgitation; AS = aortic stenosis; ASD = atrial septal defect; AVSD = atrioventricular septal defect; BAV = bicuspid aortic valve; ccTGA = congenital corrected transposition of the great arteries; CoA = coarctation of the aorta; d-TGA = dextro-transposition of the great arteries (corrected with arterial switch or a Rastelli procedure); d-TGA atrial switch = dextro-transposition of the great arteries (corrected with a Senning/Mustard procedure); DORV = double outlet right ventricle; Fontan = Fontan circulation; PA/IVS = pulmonary atresia with intact ventricular septum; PA with VSD = pulmonary atresia with VSD; PDA = patent ductus arteriosus; PS = pulmonary stenosis; ToF = tetralogy of Fallot; VSD = ventricular septal defect.

The mean aerobic exercise capacity, expressed as %EC_pred_, was 77% ± 20%. In addition, exercise capacity differed between cardiac lesions. Patients with lesions classified as moderately complex, for example, CoA, congenital aortic valve disease, and pulmonary stenosis, had a higher mean %EC_pred_ compared to patients with severely complex lesions, for example, truncus arteriosus and pulmonary atresia with VSD and Fontan circulation. There was a wide range in exercise capacity within each diagnosis (Figure 2).

### Mortality

After a mean follow-up time of 9.4 years 6.0 years, 214 patients were deceased (5.8%). These patients were older at the performed exercise test and had a lower mean %EC_pred_ compared to those who were alive. Also, the deceased patients had a higher extent of reported symptoms, had a higher NYHA functional class, had more cardiovascular medication, and had a lower self-reported physical activity level. The highest mortality rate was found among patients with simple and severely complex lesions (Supplemental Table 1).

After the performed exercise test, the estimated 20-year survival within each category of exercise capacity was 91% (≥70 %EC_pred_), 80% (50-<70 %EC_pred_), and 67% (<50 %EC_pred_) (Central Illustration).

**Central Illustration:**
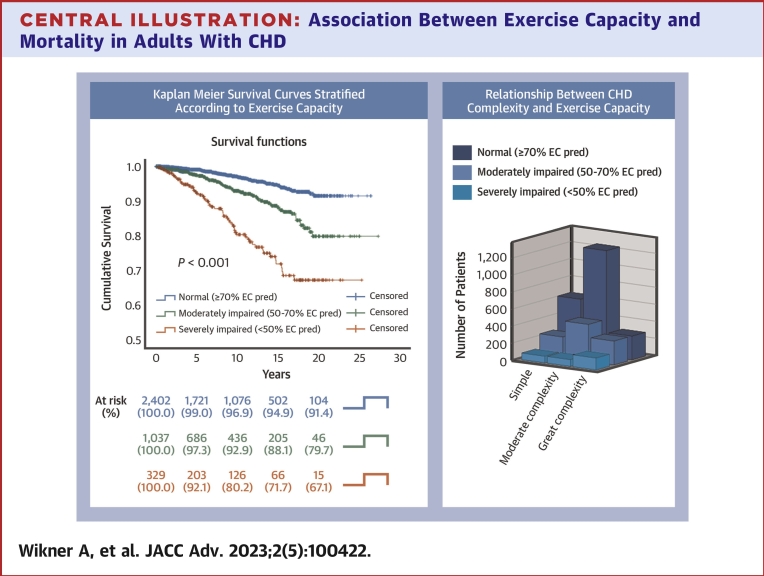
Association Between Exercise Capacity and Mortality in Adults With CHD **(Left)** Kaplan-Meier curves illustrating estimated survival among adults with congenital heart diseases (CHD) classified into 3 groups according to the percent of predicted peak exercise capacity using: log rank<0.001, %EC_pred_, and percent of predicted peak exercise capacity. **(Right)** Stacked 3-dimensional bar showing the distribution of CHD complexity according to percent of predicted peak exercise capacity, %EC_pred_, and percent of predicted peak exercise capacity.

A lower %EC_pred_ was associated with a higher risk of mortality. In the univariable Cox regression analysis, moderately impaired (50-<70 %EC_pred_) and severely impaired (<50 %EC_pred_) exercise capacity were associated with a 2 to 6 times higher risk of mortality compared to patients with a normal exercise capacity (>70 %EC_pred_) (Table 2). Furthermore, higher age at exercise test, higher NYHA class (III-IV), lower self-reported physical activity level, and having symptoms, pacemaker, cardiovascular medication, and impaired ventricular function were associated with higher mortality in a univariable Cox regression analysis (Table 2).

**Table 2:** Univariable Cox Regression Analysis Showing Factors Associated With Mortality Risk

	n	Wald	*P* Value	HR (95% CI)
Exercise capacity	3,721			
≥70%EC_pred_ ref	2,364	99.6	**<0.001**	
50-<70%EC_pred_	1,027	28.2	**<0.001**	2.3 (1.7-3.2)
<50%EC_pred_	330	98.7	**<0.001**	5.6 (4.0-7.9)
CHD complexity	3,405			
Simple	950	7.6	**0.02**	
Moderate	1,756	5.4	**0.02**	0.7 (0.5-0.9)
Great complexity	699	0.04	0.8	1.04 (0.7-1.5)
NYHA functional class	3,293			
I ref	2,635	77.9	**<0.001**	
II	549	31.1	**<0.001**	2.5 (1.8-3.5)
III-IV	109	64.3	**<0.001**	6.1 (3.9-9.6)
Male	1,658	0.6	0.4	1.1 (0.8-1.5)
Smoking	3,458			
No ref	2,915	3.3	0.2	
Previous smoker	180	1.3	0.2	1.5 (0.8-2.9)
Yes	363	2.3	0.1	1.4 (0.9-2.1)
Age at exercise test	3,721	144.7	**<0.001**	1.05 (1.04-1.06)
Cardiovascular medication^a^ yes	1,052	58.7	**<0.001**	2.9 (2.2-3.8)
Physical activity	3,372			
>3 h/wk ref	775	23.4	**<0.001**	
<3 h/wk	1,136	8.0	**0.005**	2.3 (1.3-4.0)
None	1,461	20.5	**<0.001**	3.5 (2.0-5.9)
Symptoms^b^ yes	1,096	23.9	**<0.001**	2.0 (1.5-2.6)
Pacemaker/ICD yes	152	4.9	**0.03**	1.9 (1.1-3.5)
Systemic ventricle function impaired	388	22.6	**<0.001**	2.3 (1.6-3.2)
Subpulmonary ventricle impaired	325	6.0	**0.015**	1.7 (1.1-2.6)

**Bold** values denote *P* < 0.05.

CHD = congenital heart disease; %EC_pred_ = percent of predicted peak exercise capacity; ICD = implantable cardioverter-defibrillator.

a Cardiovascular medication eg, digoxin, anticoagulant and antiplatelet therapies, anti-arrhythmia class I-IV and diuretics.

b Symptoms, ie, syncope, fatigue, palpitations, dyspnea, chest pain, and edema.

In the initial multivariable Cox regression analysis, moderately impaired (50-<70 %EC_pred_), severely impaired (<50 %EC_pred_) exercise capacity together with CHD complexity were associated with a 2 to 3 times higher risk of mortality after adjusting for sex, age, cardiovascular medication, NYHA class, physical activity (self-reported), and systemic ventricular function. This association remained in the final reduced multivariable Cox regression analysis, adjusted for age, with a 2 to 5 times higher risk of mortality (Table 3).

**Table 3:** Multivariable Cox Regression Analysis Showing Factors Associated With Risk of Mortality in Adults With CHD

	Initial Model (n = 2,609)				Final Reduced Model (n = 3,405)			
	n	Wald	*P* Value	HR (95% CI)	n	Wald	*P* Value	HR (95% CI)
Exercise capacity								
≥70 %ECpred ref	1,669	23.1	**<0.001**		2,148	78.8	**<0.001**	
50-<70 %ECpred	742	12.4	**<0.001**	2.1 (1.4-3.2)	958	22.4	**<0.001**	2.2 (1.6-3.1)
<50 %ECpred	208	21.7	**<0.001**	3.5 (2.1-6.0)	299	78.5	**<0.001**	5.4 (3.7-7.9)
CHD complexity								
Simple ref	690	10.1	**0.006**		950	17.7	**<0.001**	
Moderate	1,351	8.7	**0.003**	1.9 (1.2-3.0)	1,756	7.5	**0.006**	1.6 (1.2-2.3)
Great complexity	568	7.1	**0.008**	2.3 (1.3-4.2)	699	17.4	**<0.001**	2.6 (1.7-4.2)
NYHA functional class								
I ref	2,094	3.2	0.2					
II	428	0.3	0.6	1.1 (0.7-1.7)				
III-IV	87	3.1	0.08	1.7 (0.9-2.9)				
Sex								
Man	1,479	0.1	0.7	0.9 (0.7-1.3)				
Cardiovascular medication^a^								
Yes	710	0.4	0.5	1.1 (0.8-1.7)				
Physical activity								
>3 h/wk ref	573	2.1	0.3					
<3 h/wk	852	1.4	0.2	1.5 (0.8-3.1)				
None	1,184	2.1	0.1	1.7 (0.8-3.3)				
Systemic ventricular function								
Impaired	304	2.1	0.2	1.4 (0.9-2.3)				

Both models are adjusted for age. Number of deceased patients in initial model; n = 143 and final reduced model; n = 199. **Bold** values denote *P* < 0.05.

CHD = congenital heart disease; %EC_pred_ = percent of predicted peak exercise capacity.

a Cardiovascular medication, EG, digoxin, anticoagulant and antiplatelet therapies, anti-arrhythmia class I-IV and diuretics.

The use of %ECpred in predicting risk of mortality has an additive value. When adding %ECpred to the analyses, the AUC in the initial model increases from 0.81 to 0.83 at 5 years and 0.81 to 0.82 at 10 years. For the final reduced model, the corresponding values were 0.77 to 0.80 and 0.76 to 0.80.

### Characteristics of the patients within categories of exercise capacity

**Figure 3:**
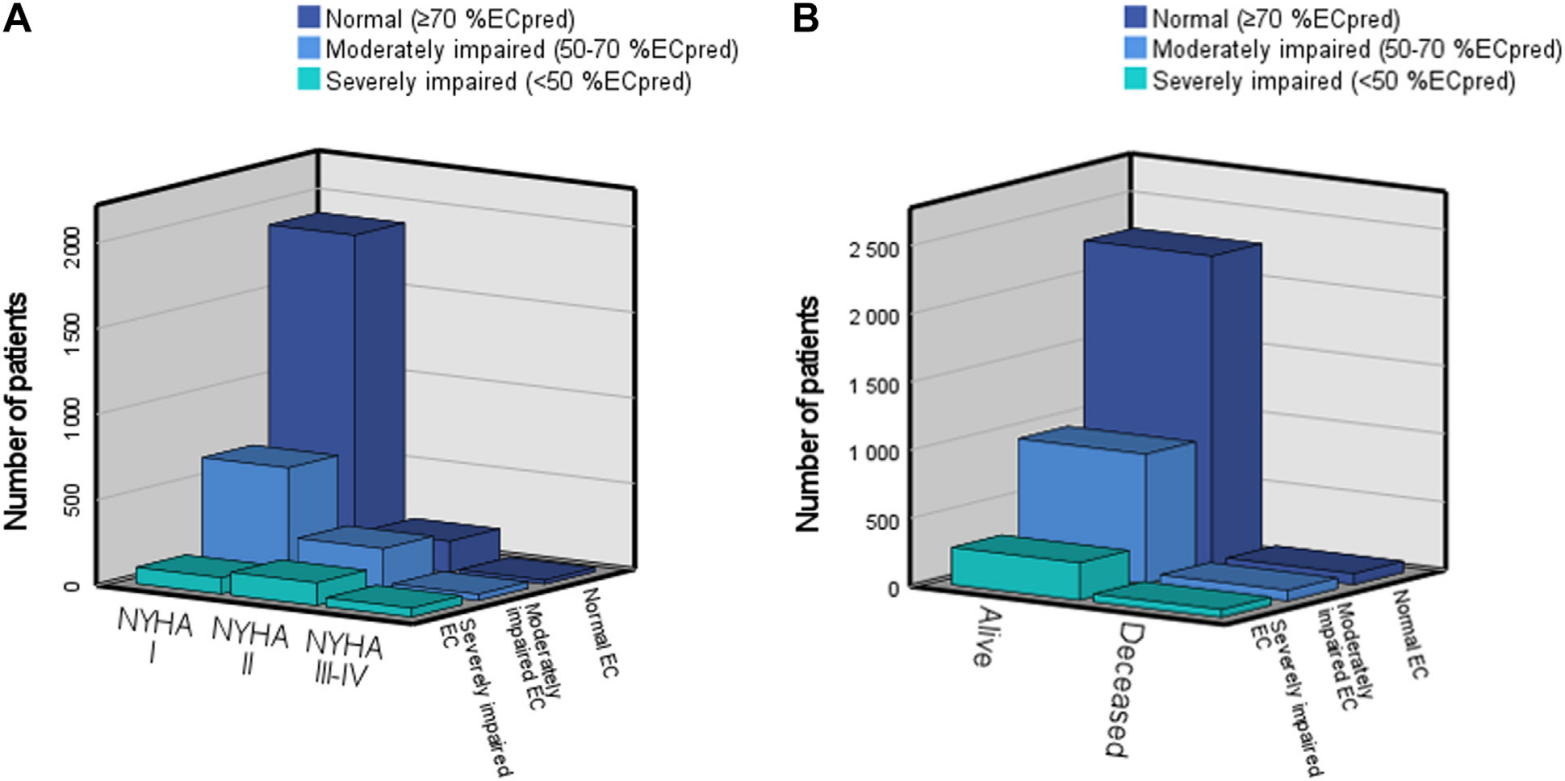
**Distribution of NYHA Functional Class and Deceased Patients According to Percent of Predicted Exercise Capacity** Stacked 3-dimensional bar showing the distribution of **(A)** NYHA functional class and **(B)** deceased patients according to percent of predicted peak exercise capacity (%EC_pred_).

CHD complexity and NYHA class were represented in all 3 categories of exercise capacity, that is normal, moderately, and severely impaired (Central Illustration, Figure 3A). Furthermore, these 3 categories of exercise capacity were evenly distributed among deceased patients (normal, n = 80 [37%]; moderately impaired, n = 77 [36%]; and severely impaired, n = 57 [27%]) (Figure 3B). However, the mortality rate was highest in patients with impaired exercise capacity (<70%EC_pred_). There was no difference between men and women regarding exercise capacity categories (data not shown).

In a dropout analysis, comparing patients with data on exercise capacity (included) with those without data on exercise capacity (excluded), the mortality rate was higher within the excluded group (214 [5.8%] deaths in 3,721 patients vs 551 [7.2%] deaths in 7,699 patients, *P* ≤ 0.005). Furthermore, when analyzing mortality in relation to years of observation, in the group of patients with data on exercise capacity there were 521 deceased per 100,000 patient years compared to 995 deceased per 100,000 patient years in the group with no data on exercise capacity. Also, the median age at death was higher within the group of excluded patients (60.6 years vs 52.4 years) and most deaths occurred in patients with simple lesions, ie, atrial septal defect (36.3%). In addition, there were more men in the included group (n = 2,063 [55.4%] vs n = 3,716 [45.5%], *P* < 0.001) and data on exercise capacity were at a higher extent present in patients with more complex lesions^6^ (Supplemental Figure 1).

## Discussion

To the best of our knowledge, this multicenter study is the largest of its kind (patient population, n = 3,721) examining the association between aerobic exercise capacity and mortality in patients with various severity of CHD. We found that impaired exercise capacity (<70 %EC_pred_) was associated with a 2 to 5 times higher risk of death, demonstrating that peak exercise capacity can be a useful tool to identify patients at risk.

The large patient population allowed us to examine mortality as a primary endpoint. Previous reports are either from single center with composite endpoints^8^ or are based on subgroups of CHD diagnoses.^14^^,^^16^^,^^17^ In contrast to previous reports by Inuzuka et al^15^ and Diller et al,^8^ the number of patients with simple lesions was larger in the present report. Differences in populations are likely due to the inclusion of data from multiple centers in our study leading to a larger study population with more diverse CHD severity, thus increasing the generalizability. Although generalizability may be better, the results of the dropout analysis indicate that exercise tests are more likely to be performed in patients with more complex lesions. Previous studies have shown that even in less complex lesions such as CoA,^22^ valvular disease, and atrioventricular septal defect, mortality is still higher when compared to the general population.^4^ This suggests that regular evaluation of exercise capacity in all patients with CHD may be useful, not just in patients with more complex lesions.^23^^,^^24^

Patients with impaired exercise capacity (<70%EC_pred_) had the highest mortality, especially patients with severely impaired exercise capacity (<50%EC_pred_). The group of patients includes patients with various CHD lesion complexity and NYHA class. Nevertheless, based on the present data, more impaired exercise capacity in combination with greater lesion complexity is associated with the higher risk of mortality. It is important to highlight that including exercise capacity in the model increases the AUC. In addition, exercise capacity in a reduced model performs similar. Thus, exercise capacity has an additive value when used together with other variables, but most importantly, is very powerful in a parsimonious model including age and complexity of heart lesion.

The median age at death in the present study was 52.7 years (IQR: 35.2-66.9 years), which is in similar to the study by Goldstein et al^25^ (64.2 years). In contrast, Oechslin et al^26^ reported a mean age at death of 37 ± 15 years in a similar subset of patients as in the present study. Differences in the age at death in our study compared to the latter may be due to improvements in survival since 2000. Previous studies have reported on increased survival among patients with CHD during the last decades.^1^^,^^3^^,^^27^

It has been shown that NYHA functional class is associated with mortality.^28^ This is similar to the univariable Cox regression results in our study. However, this association was not significant in the multivariable model. Therefore, NYHA functional class may not be as reliable as an objective measure of exercise capacity (%EC_pred_) when estimating risk of mortality.

In Scandinavia, peak exercise tests on cycle ergometers are commonly used in the clinical setting with peak workload as the primary outcome. Whereas, cardiopulmonary exercise testing with peak oxygen uptake as the main outcome is more commonly used internationally. Yet, it is well-known that these variables are highly correlated.^29^ Despite differences in test modalities, our findings of gradually declining exercise capacity with increasing CHD complexity are in line with previous reports, yet herein presented with a larger patient population.^7^^,^^8^^,^^13^^,^^15^ Also, the wide range in exercise capacity within each diagnosis and the overlap between groups of diagnoses regarding exercise capacity confirm previous reports.^7^^,^^8^^,^^13^^,^^15^ This further stresses the need to evaluate the exercise capacity of the individual patient, instead of making decisions based on diagnosis and structural heart lesion alone.

### Study limitations

A known limitation of registry studies is missing data, which may complicate analyses. In the present report, data on height were missing in 139 patients. Therefore, height was imputed to enable calculation of the predicted peak exercise capacity. A post hoc analysis of percent of predicted peak exercise capacity between populations with and without imputed data on height showed no difference (data not shown). A second limitation might be that the exercise tests were performed over a long-time span meaning that slightly different test protocols have been used, that is, the initial workload and the incremental increase of workload may differ. Yet, the effect of different protocols on the achieved peak exercise capacity is most likely limited and probably insignificant in view of the large study population. Furthermore, we cannot rule out that some of the exercise tests were performed in proximity to an intervention, and therefore some tests might not reflect exercise capacity in a clinically stable situation. Nevertheless, due to large numbers of tests, we deem it unlikely that this impacted the overall results. Moreover, the dropout analysis showed that the mortality was higher among patients lacking data on exercise capacity, which contradicts that included patients would be in a worse condition. As noted previously, exercise tests are less likely to be performed in patients with lesions classified as simple. Even if mortality is still higher in this group compared to the general population, mortality is lower compared with more severe cardiac lesions. Therefore, this bias may cause an underestimation of the importance of exercise capacity in predicting mortality.

## Conclusions

This large multicenter study demonstrates that impaired exercise capacity is associated with all-cause mortality in adults with CHD. Exercise capacity <70% of predicted, together with increased disease complexity, were independently associated with a higher mortality. Evaluation of exercise capacity is an easily accessible tool for risk assessment in adults with CHD.

PERSPECTIVES **COMPETENCY IN PATIENT CARE:** Impaired exercise capacity (<70% of predicted) is common, and together with lesion complexity, is independently associated with all-cause mortality in adults with CHD. Exercise testing should be implemented in the regular follow-up of all patients with CHD in order to identify patients at risk of worse outcome, regardless of cardiac lesion.

**TRANSLATIONAL OUTLOOK:** Evaluation of exercise capacity is an easily accessible tool for risk assessment in adults with CHD, although confirmation in prospective studies is needed.

## Funding support and author disclosures

This study is supported by grants from the 10.13039/501100003793Swedish Heart-Lung Foundation (20100355, 20130472, 20170483, 20190525, 20200493), the research foundation of health care professions within cardiology, 10.13039/501100004885Umeå University, 10.13039/501100014689Region Västerbotten (the County of Västerbotten), Heart Foundation of Northern Sweden and Visare Norr. The authors have reported that they have no relationships relevant to the contents of this paper to disclose.
