# Supplementary material for: Impaired Exercise Capacity and Mortality Risk in Adults With Congenital Heart Disease
Source: JACC Adv. 2023 Jul 28;2(5):100422. doi: 10.1016/j.jacadv.2023.100422 (PMC11198387; doi:10.1016/j.jacadv.2023.100422)
Supplement: Supplemental Figure 1 and Table 1 [file mmc1.pdf]

Online appendix

Page **Title**

2 **Online Figure 1.** Distribution of diagnosis between groups of included and excluded patients.

3-5 **Online Table 1.** Exercise capacity, death and NYHA class according to diagnosis

Online Figure 1. Distribution of diagnosis between groups of included and excluded patients.

Stacked bar chart showing the distribution of diagnosis between included and analysed patients in comparison to excluded and not analysed patients.

AS, aortic stenosis; AR, aortic regurgitation; BAV, bicuspid aortic valve; CoA, coarctation of the aorta, PS, pulmonary stenosis; ASD, atrial septal defect; PDA, patent ductus arteriosus; VSD, ventricular septal defect; d-TGA, dextro-transposition of the great arteries (corrected with arterial switch or a Rastelli procedure); ToF, tetralogy of Fallot; PA/IVS, pulmonary atresia with intact ventricular septum; DORV, double outlet right ventricle; AVSD, atrioventricular septal defect; ccTGA, congenital corrected transposition of the great arteries; d-TGA atrial switch, dextro-transposition of the great arteries (corrected with a Senning/Mustard procedure); PA with VSD, pulmonary atresia with VSD; Fontan, Fontan circulation.

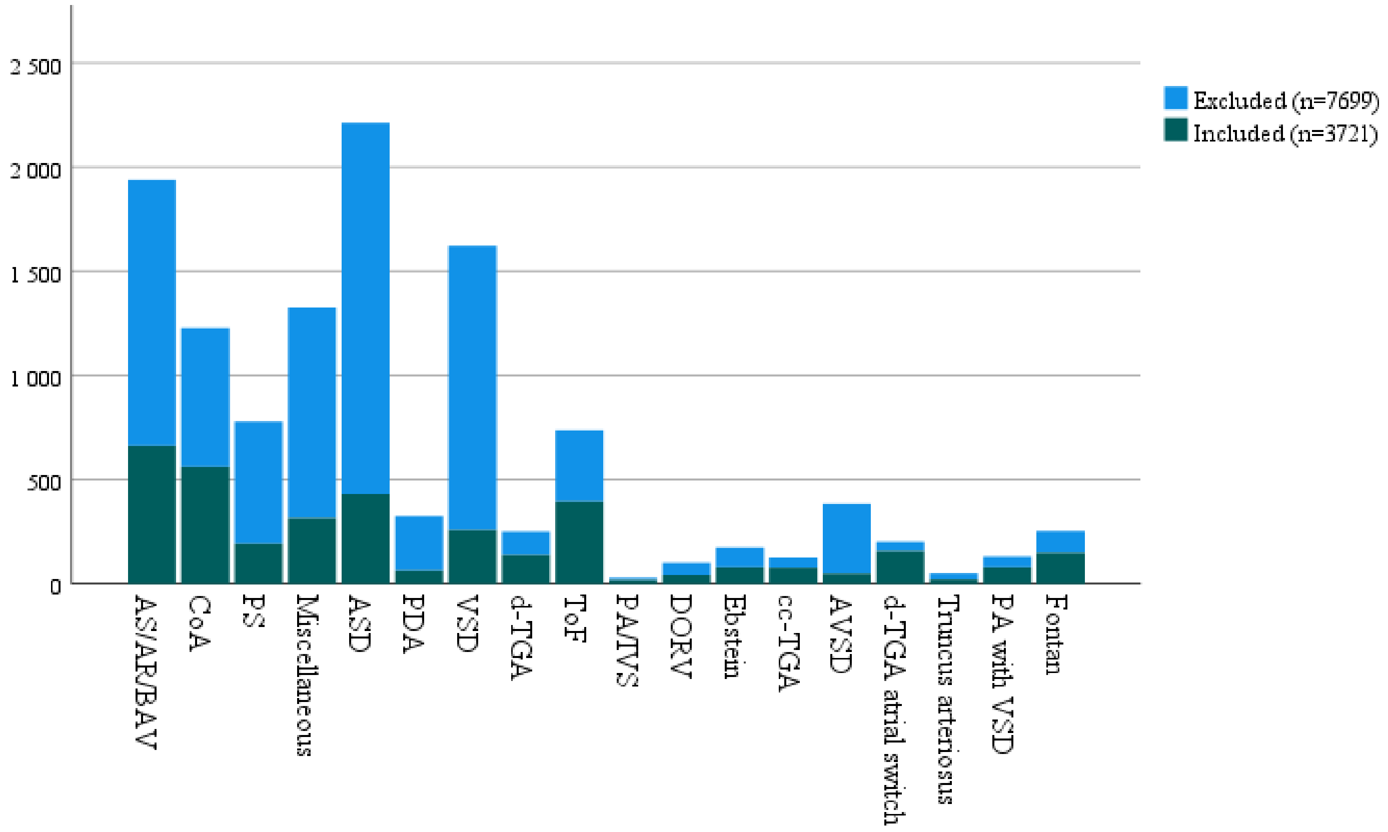

2 500
2 000
1 500
1 000
500
0
Excluded (n=7699)
Included (n=3721)
AS/AR/BAV
CoA
PS
Miscellaneous
ASD
PDA
VSD
d-TGA
ToF
PA/IVS
DORV
Ebstein
cc-TGA
AVSD
d-TGA atrial switch
Truncus arteriosus
PA with VSD
Fontan

Online Table 1. Exercise capacity, death and NYHA class according to diagnosis

**Diagnosis**	**All** *n*=3721	**%EC$_{pred}$**	**Age at exercise test**	**Deceased per diagnosis group (*n*=214)**	**Age at death**	**NYHA class I/ II/ III/IV (%) (*n*=3293)**
**AS/AR/BAV**	664 (17.8)	83.5±18.4	27.9 (21.0, 39.0)	26 (3.9)	48.5 (35.8, 56.5)	90/8/2/0
**CoA**	562 (15.1)	83.8±17.9	27.1 (21.0, 40.0)	17 (3.0)	57.0 (38.2, 65.1)	95/4/1/0
**PS**	194 (5.2)	79.9±22.1	29.96 (22.1, 46.4)	9 (4.6)	56.9 (37.5, 64.9)	76/22/2/0
**Miscellaneous**	316 (8.5)	77.8±20.6	26.2 (20.5, 38.0)	15 (4.7)	33.2 (24.4, 47.8)	82/15/3/0
**ASD**	432 (11.6)	79.4±21.8	46.4 (32.0, 60.5)	41 (9.5)	74.7 (68.4, 79.2)	67/26/7/0
**PDA**	64 (1.7)	79.4±21.2	39.2 (27.4, 54.7)	7 (10.9)	70.3 (51.2, 78.4)	62/34/4/0
**VSD**	260 (7.0)	77.9±20.9	31.1 (22.6, 44.9)	12 (5.6)	65.8 (51.3, 82.9)	82/17/1/0
**d-TGA**	139 (3.7)	75.2±19.4	20.6 (19.2, 23.0)	9 (6.5)	27.4 (24.0, 30.1)	86/12/2/0

**ToF**	397 (10.7)	73.2±17.8	25.3 (20.6, 36.5)	29 (7.3)	53.0 (46.1, 60.5)	82/16/2/0
**PA/IVS**	21 (0.6)	72.0±20.9	20.4 (18.8, 25.1)	0 (0.0)	-	74/21/5/0
**DORV**	44 (1.2)	71.4±16.7	21.2 (19.5, 25.3)	1 (2.3)	29.1	83/14/3/0
**Ebstein**	82 (2.2)	71.0±19.4	40.2 (23.9, 50.6)	9 (11.0)	55.8 (41.9, 66.4)	64/32/4/0
**cc-TGA**	79 (2.1)	71.5±23.5	29.6 (20.6, 42.3)	7 (8.9)	53.8 (30.6, 61.6)	64/28/8/0
**AVSD**	51 (1.4)	68.5±16.6	21.2 (19.5, 25.2)	2 (3.9)	40.8	70/28/2/0
**d-TGA atrial switch**	159 (4.3)	68.5±17.3	22.9 (19.9, 29.9)	9 (5.7)	35.7 (28.9, 48.6)	74/24/2/0
**Truncus arteriosus**	24 (0.6)	64.7±19.8	21.3 (19.8, 25.0)	1 (4.2)	47.7	65/25/10/0
**PA with VSD**	82 (2.2)	60.7±17.1	20.9 (19.8, 25.9)	7 (8.5)	33.5 (30.6, 44.0)	64/27/9/0
**Fontan**	151 (4.1)	56.3±14.6	20.4 (19.1, 22.6)	13 (8.6)	39.5 (29.2, 47.8)	55/38/7/0

Data are presented as n (%), mean (±SD), and median (25th, 75th quartile).

$\%EC_{pred}$, percent of predicted peak exercise capacity; NYHA, New York Heart Association; AS, aortic stenosis; AR, aortic regurgitation; BAV, bicuspid aortic valve; CoA, coarctation of the aorta, PS, pulmonary stenosis; ASD, atrial septal defect; PDA, patent ductus arteriosus; VSD, ventricular septal defect; d-TGA, dextro-transposition of the great arteries (corrected with arterial switch or a Rastelli procedure); ToF, tetralogy of Fallot; PA/IVS, pulmonary atresia with intact ventricular septum; DORV, double outlet right ventricle; AVSD, atrioventricular septal defect; ccTGA, congenital corrected transposition of the great arteries; d-TGA atrial switch, dextro-transposition of the great arteries (corrected with a Senning/Mustard procedure); PA with VSD, pulmonary atresia with VSD; Fontan, Fontan circulation.
